# Design, Manufacture, Test and Experiment of Six-Axis Force Torque Sensor for Chinese Experimental Module Manipulator

**DOI:** 10.3390/s22093603

**Published:** 2022-05-09

**Authors:** Yongjun Sun

**Affiliations:** 1 State Key Laboratory of Robotics and System, Harbin Institute of Technology, Harbin 150001, China; sunyongjun@hit.edu.cn; 2 School of Mechatronics Engineering, Harbin Institute of Technology, Harbin 150001, China

**Keywords:** space manipulator, six-axis force/torque sensor, design, manufacture, test, experiment

## Abstract

A novel six-axis force/torque sensor (F/T sensor) for an Experimental Module Manipulator (EMM) in the Chinese Space Station (CSS) is developed in this paper. First, we designed the elastomer structure of the F/T sensor and used the analytical method and the finite element method to analyze the strain, in order to accomplish the strain gauges’ layout. Then, the electrical system was designed, which mainly realizes the acquisition of force/torque information, temperature and serial communication with the end effector (EE). Following this, we analyzed and designed the adaptability of the F/T sensor to the space environment. After this, the manufacturing process of the sensor was introduced in detail, and the F/T sensor was calibrated by a pulley weight system. Finally, the sensor was tested on the space environment adaptability of mechanical vibration and thermal vacuum on the ground. The test results show that the developed sensor has the ability to accurately measure three-dimensional force and three-dimensional moment information on orbit, which provides necessary conditions for the on-orbit fine operation of EMM.

## 1. Introduction

The Chinese Space Station (CSS) will be constructed in 2022. It includes one core module (CM), two experimental modules (EM) and one cargo spaceship [1]. The space manipulators will play significant roles in completing space tasks, such as on-orbit assembly, maintenance, manipulation assistance, payload care and astronaut on-orbit support. So far, these space manipulators have been developed for the International Space Station (ISS). They are the Space Station Remote Manipulator System (SSRMS), designed by the Canada Space Agency (CSA), the Japanese Experimental Module Remote Manipulator System (JEMRMS), devised by the Japan Aerospace Exploration Agency (JAEA), and the European Robotic Arm (ERA), developed by the European Space Agency (ESA) for the Russian module [2,3,4]. Moreover, the space manipulator for the CM of the CSS launched in 2021.

The existing space manipulators have an obvious significant feature—all of them equipped the six-axis force/torque sensor (F/T sensor). This sensor could measure the force/torque information when the end effector contacts the environment or grasps payloads in the space station. Taking advantage of the force/torque information, the space manipulator will complete a series of complicated and fine work, and consequently, realize the intelligence.

The CSS has two space manipulators, with the big one installed on the CM and the small one will be located on the EM. The small manipulator, namely Experimental Module Manipulator (EMM), which can work independently on the EM to look after exposed experimental and optical platforms, checks the modules and accomplishes EVA support. For enhancing the force control capability of the space manipulator, the F/T sensor should be configured. The F/T sensor is an important part, which is used to sense the contact multi-dimensional force/torque information during the EMM’s operating load process, and provide a basis for the EMM’s impedance control or compliance force control. If the space manipulator does not have the F/T sensor, it will greatly weaken the capability of the space manipulator when it is necessary to interact with the environment. Different from the common F/T sensor on the ground [5,6,7,8,9,10,11,12], the F/T sensor of the EMM will be subjected to harsh effects, such as mechanical vibration, cosmic radiation, high vacuum, microgravity, and drastic temperature changes. In this paper, an F/T sensor is developed for EMM.

## 2. Space Manipulator and F/T Sensor

**Figure 1:**
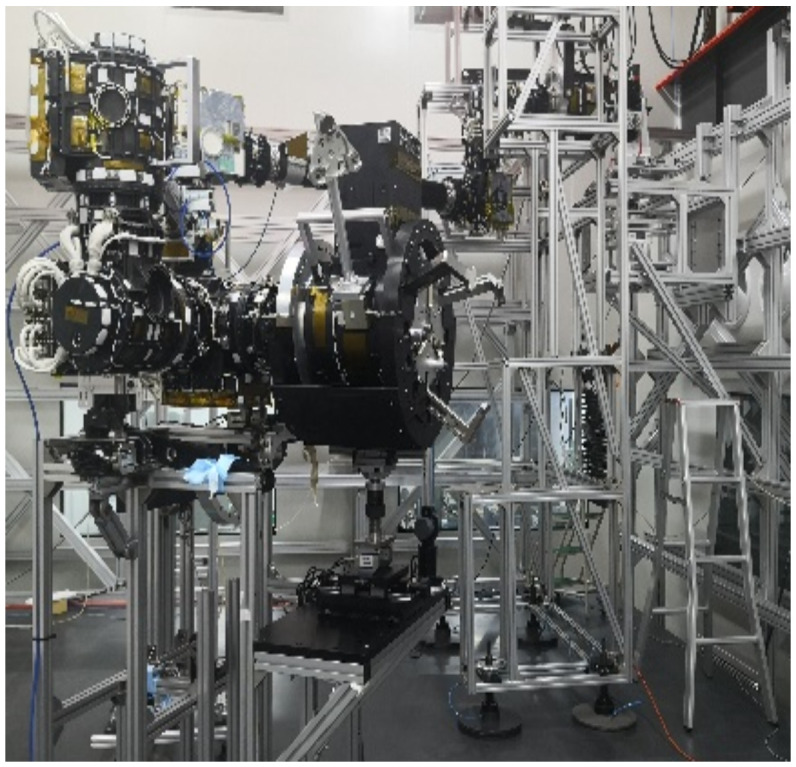
Space manipulator.

**Figure 2:**
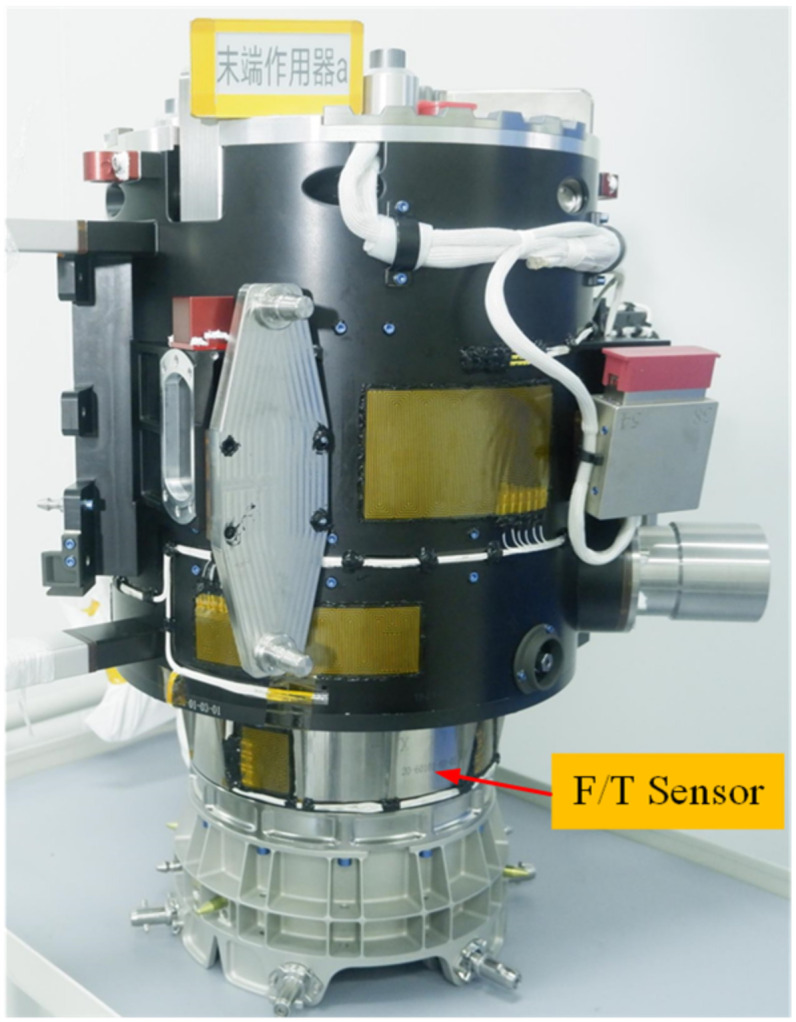
F/T sensor on EE.

The EMM, shown in Figure 1, is a 7 DOF manipulator. It includes two wrist joints (6 DOF), one elbow joint (1 DOF), two rods, two F/T sensors and two end effectors (EE). The EMM is 5 meters long when it is unfolded. The F/T sensor fixed between the end effector and modular interface, is shown in Figure 2.

## 3. F/T Sensor Design

### 3.1. Measuring Principle

The F/T sensor uses resistance strain gauges to detect small deformations [13,14,15,16,17]. Resistance-strain-testing technology is a very mature technology in the field of force/moment/torque sensors. Under the action of an external load, a strain gauge is used to convert the small elastic deformation of the metal into a change in the resistance of the strain gauge, and a Wheatstone bridge is applied to convert the resistance change into a voltage or current signal, thereby realizing the conversion of the difficult-to-measure non-electricity information into an easy-to-measure electricity signal.

### 3.2. Sensor Structure Design

**Figure 3:**
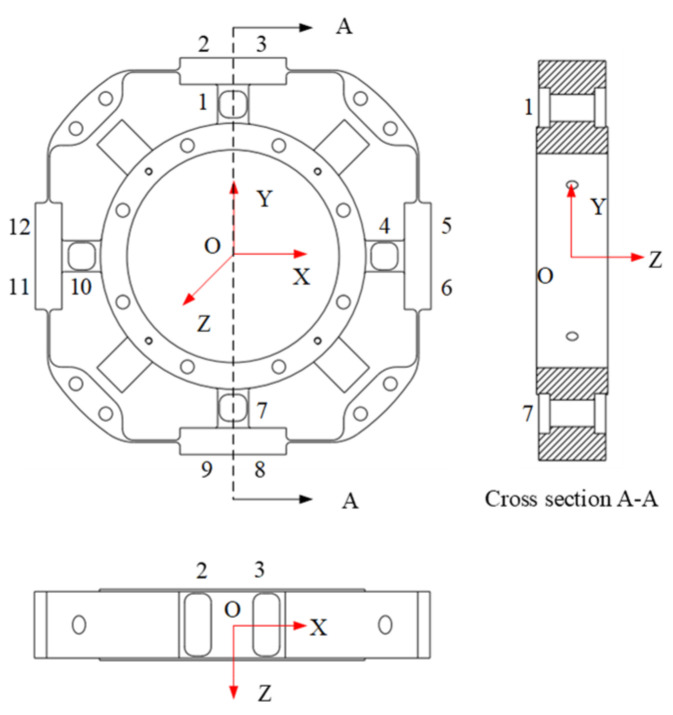
Sensor model.

Considering the performance of sensors and aerospace products, titanium alloy material Ti-6Al-4V was selected. It is a monolithic elastic element, and the structure is symmetrical. The sensor model is shown in Figure 3.

### 3.3. Strain Analysis Based on Analytical Method

Strain distribution is essential for the design of F/T sensors, for which the analytical method is the common method [18,19,20,21].

#### 3.3.1. Fx/Fy/Fz Force Analysis

**Figure 4:**
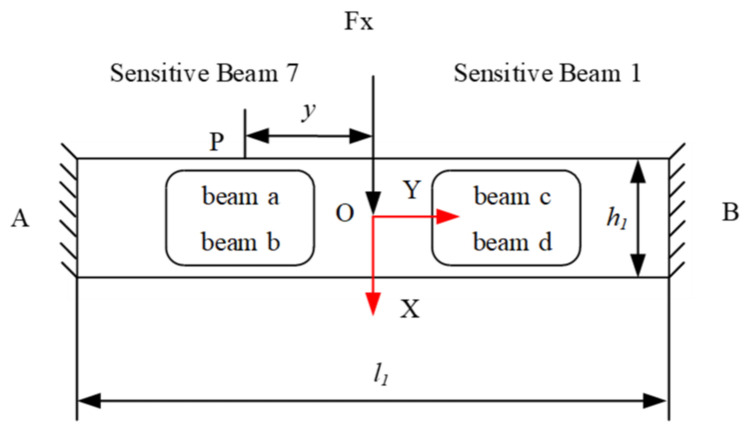
Free body diagram of the sensitive beams for *Fx.*

Figure 4 shows the free body diagram of the plate beams for the *Fx* sensor or the *Fy* sensor under the force of *Fx* or *Fy*. It can be seen that, in Figure 3, sensitive beam 1 and 7 are symmetrical on the vertical axis (X-axis), and sensitive beam 1 and 7 are symmetrical on the horizontal axis (Y-axis), and also sensitive beam 4 and beam 10 are symmetrical on the horizontal/vertical axis. Thus, equations are used for analyzing the strains inferred on the upper and the lower surfaces of beam 1, and these may applied to beam 7, 4 and 10. The equations under the force *Fx* may be applied to sensitive beam 4 and 10 for the *Fy* sensor, because sensitive beam 1 and 7, and 4 and 10 are the same in the structure.

**Figure 5:**
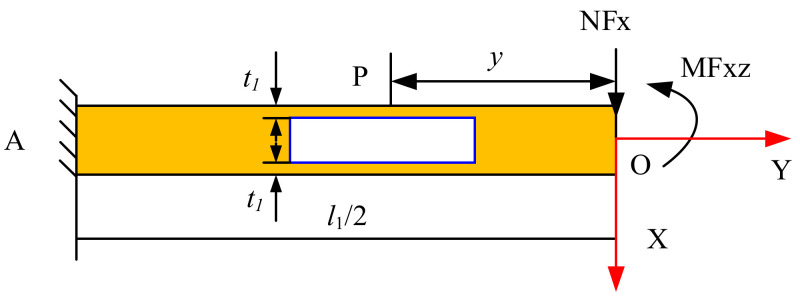
Free body diagram of the plate beams.

According to the theory of mechanics, the loading forces at points A and B can be known. Taking point A as the research object, *M*_A_ = *l*_1_/8 and *NF*_A_ = *Fx*/2. Taking the OA segment as the research object for force analysis, the force diagram is shown in Figure 5.

From the force balance relationship at point A, the moment equilibrium condition is $\sum M_{A}=0$ [22].

*NFx* = *Fx*/2 (1)

(2) $$M_{A}+MFxz-NFx\frac{l_{1}}{2}=0$$

Therefore,

*MFxz* = *Fxl*_1_/8 (3)

**Figure 6:**
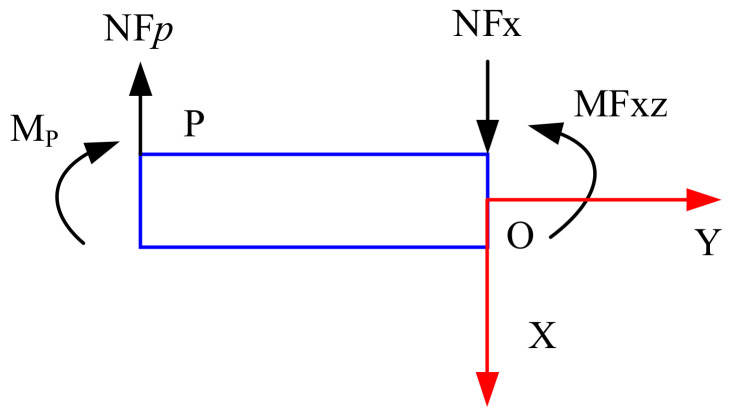
Free body diagram of the plate beams for *Fz.*

Take any point P on sensitive beam 6, and the force analysis is shown in Figure 6.

It can be seen from the moment balance at point P, the moment equilibrium condition is $\sum M_{P}=0$ [22].

(4) $$M_{P}+MFxz-NFx\cdot y=0$$

Therefore,

(5) $$M_{P}=NFx\cdot y-MFxz=\frac{Fx}{2}(y-\frac{l_{1}}{4})=0$$

Therefore, the strain at any point P on sensitive beam 7 is

*ε* = *M*_P_/*EI*_Z_ (6)

According to Figure 5 and Figure 6, *I_Z_* can be obtained from the principle of the superposition method in mechanics

*I*_Z_ = *I*_Z1_ − *I*_Z2_ = 1/6 *b*_1_*t*_1_(3*h*_1_^2^ − 6*h*_1_*t*_1_ + 4*t*_1_^2^) (7)

Substituting Equations (5) and (7) into (6), the strain on any point P on sensitive beam 7 can obtained as

(8) $$\varepsilon_{P}=\frac{3Fx(y-l_{1}/4)}{Eb_{1}t_{1}(3h_{1}{}^{2}-6h_{1}t_{1}+4t_{1}{}^{2})}$$

#### 3.3.2. Mx/My/Mz Moment Analysis

**Figure 7:**
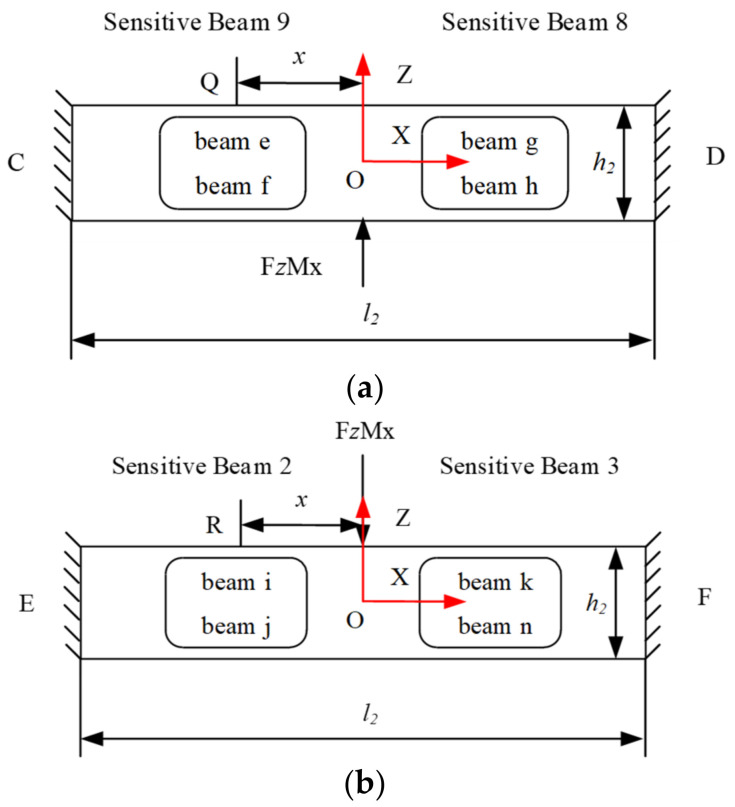
Free body diagram of the plate beams for *Fz.* (**a**) Free body diagram of the sensitive beam 8 and beam 9; (**b**) Free body diagram of the sensitive beam 2 and beam 3.

Taking *Mx* as the object and performing strain analysis, the force analysis under *Mx* can be seen, as shown in Figure 7. *Mx* can be equivalent to a pair of force couples. Assuming that the force arm is *d*, the force acting on sensitive beams 9 and 8, and sensitive beams 2 and 3 is

*FZMx* = *Mx/d* (9)

Therefore, similar to the *Fx*, the strain of Q and R at any point can be obtained as

(10) $$\varepsilon_{Q}=\frac{3Mx(x-l_{2}/4)}{Eb_{2}t_{2}(3h_{2}{}^{2}-6h_{2}t_{2}+4t_{2}{}^{2})d}$$

(11) $$\varepsilon_{R}=-\frac{3Mx(x-l_{2}/4)}{Eb_{2}t_{2}(3h_{2}{}^{2}-6h_{2}t_{2}+4t_{2}{}^{2})d}$$

### 3.4. Strain Analysis by Finite Element Method

Fx Strain Analysis

Compared with the analytical methods, finite element methods have higher solution accuracy and are widely used in engineering applications.

**Figure 8:**
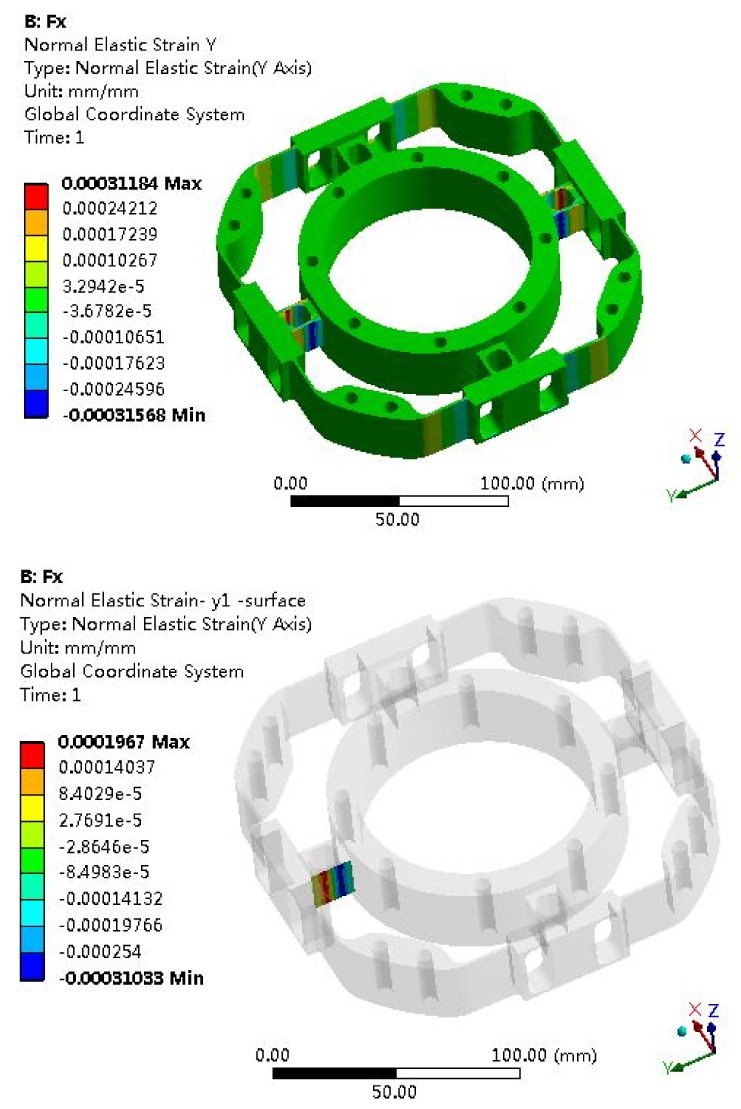
Strain cloud map under *Fx* = 500 N.

**Figure 9:**
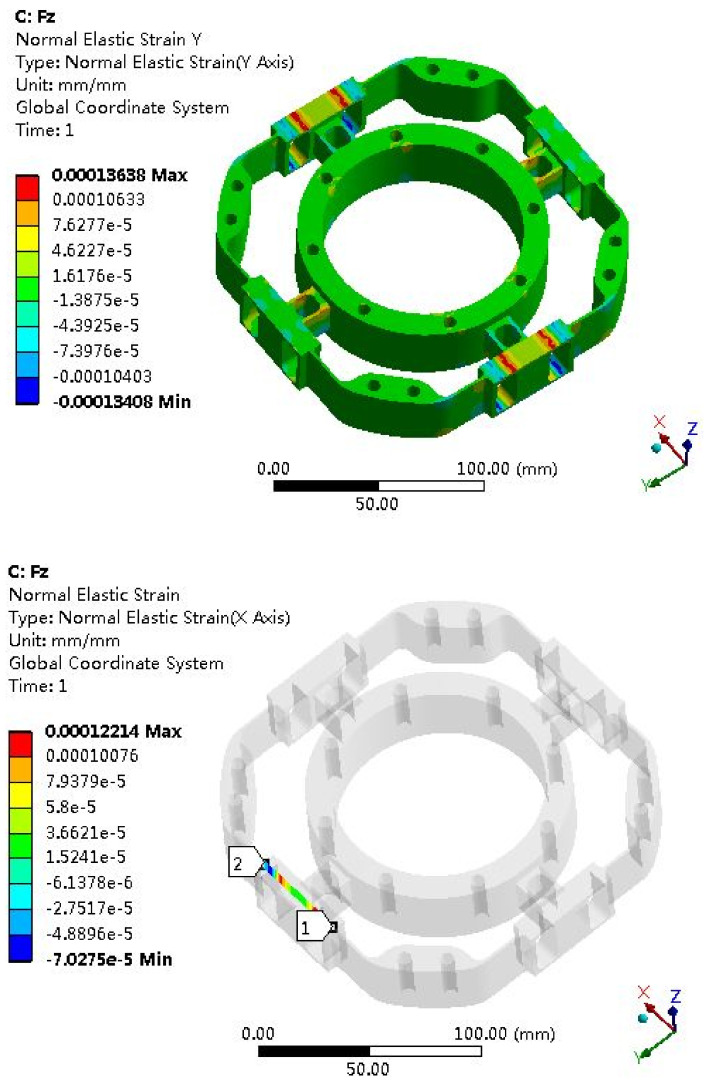
Strain cloud map under *Fz* = 500 N.

**Figure 10:**
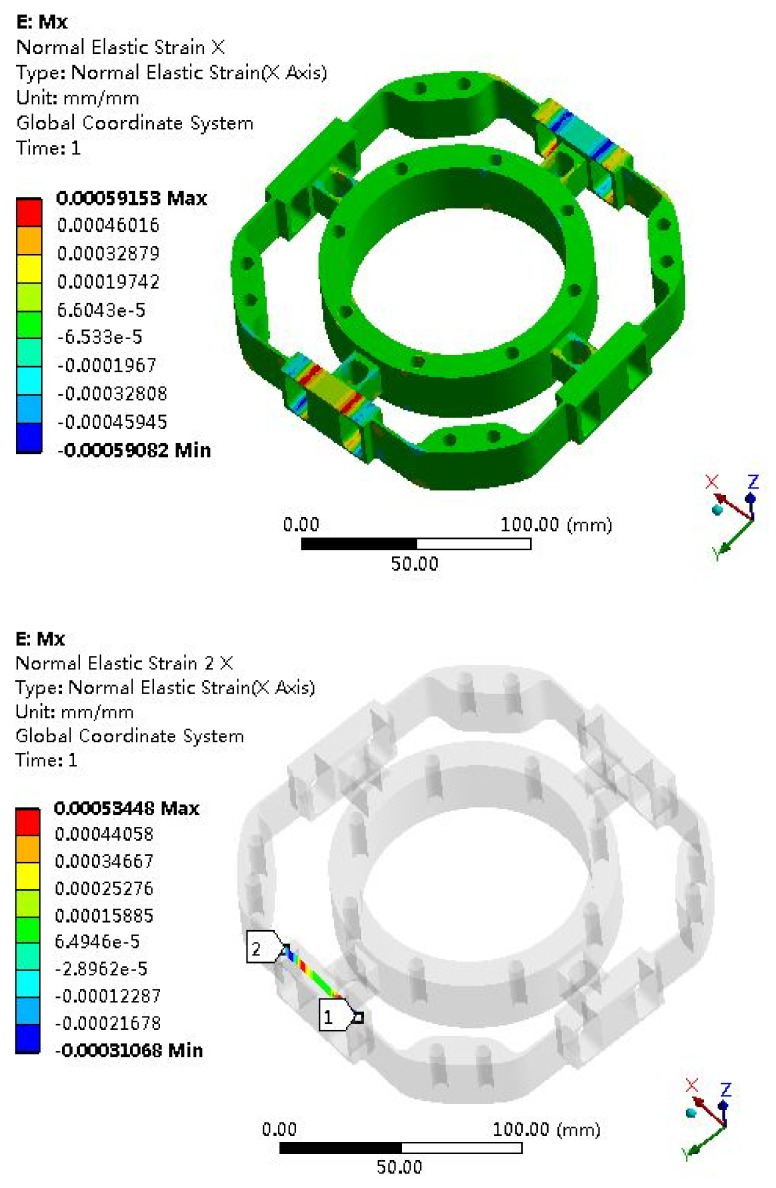
Strain cloud map under *Mx* = 100 Nm.

**Figure 11:**
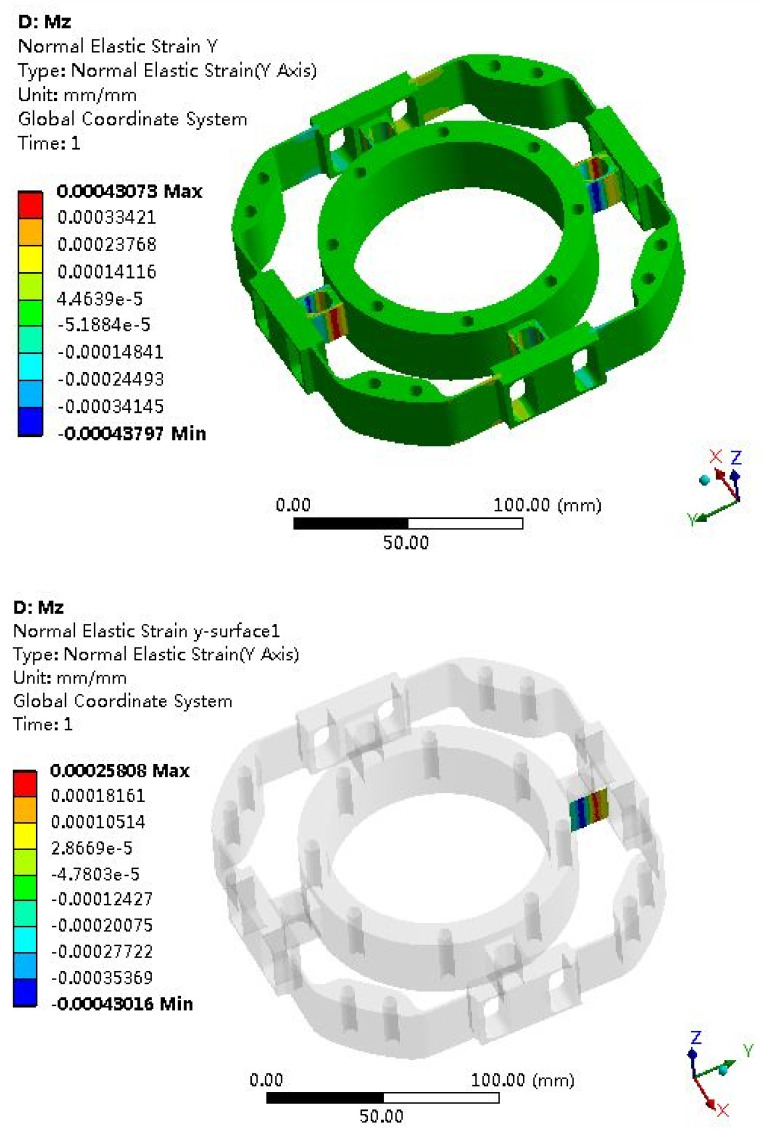
Strain cloud map under *Mz* = 100 Nm.

The elastomer of the F/T sensor plays an important role, and the structural optimization of the elastomer usually adopts the finite element method. Due to the symmetry of the elastomer, it can be seen as four cases: *Fx* (*Fy*), *Fz*, *Mx* (*My*) and *Mz*. The strain clouds are shown in Figure 8, Figure 9, Figure 10 and Figure 11.

Fz Strain Analysis

Mx Strain Analysis

Mz Strain Analysis

### 3.5. Strain Gauges Selection and Arrangement

**Figure 12:**
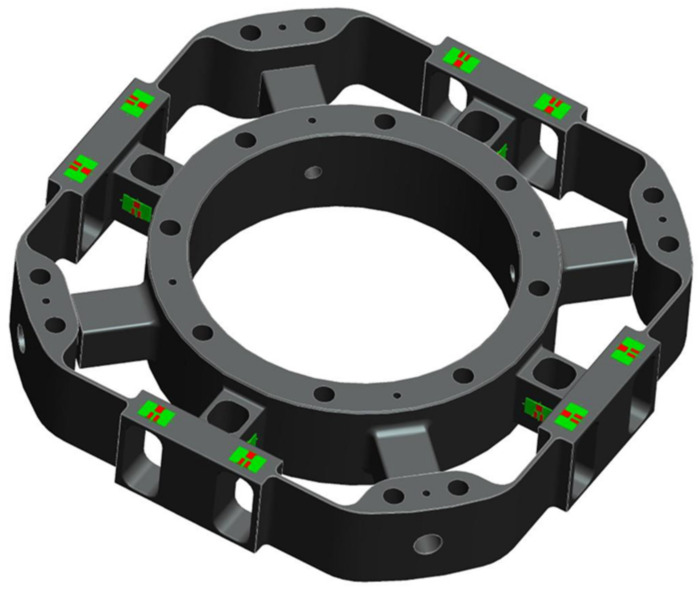
Position distribution of strain gauges.

We selected a high-precision strain gauge; the model is TK-05-T011Q-350/DP and the manufacturer is Micro-Measurements of Vishay Precision Group. According to the finite element simulation results for the elastomer, the pasting positions of strain gauges can be seen and are shown in Figure 12. Thirty-two strain gauges are pasted on the F/T sensor. Moreover, each four-strain gauge forms a full bridge, so eight full bridges are formed.

### 3.6. Sensor Electrical System Design

**Figure 13:**
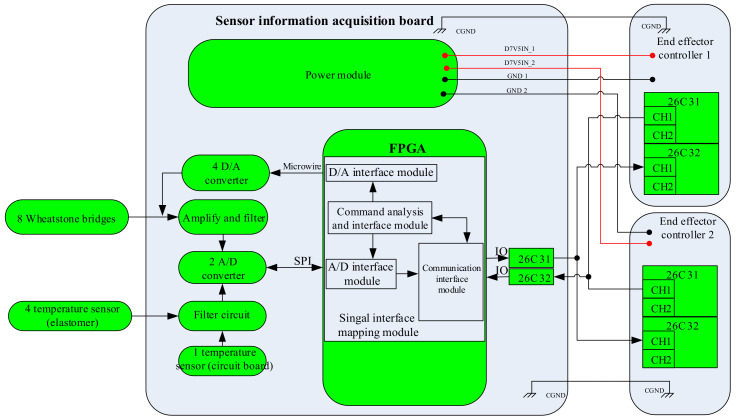
Block diagram of the electrical system in the F/T sensor.

The block diagram of the electrical system for the F/T sensor is shown in Figure 13. It shows the complete AD acquisition of force/torque information, temperature (elastomer and circuit board) and analog reference voltage, DA setting output and RS422 serial communication. It includes the following functional modules:

(1) Power module. After filtering the digital 7.5 V power provided by the controller circuit, the voltage is converted into digital 5 V; then, the digital 5 V voltage is converted into digital 2.5V and analog 5V; finally, the analog 5V is converted into analog 2.5 V;
(2) The communication interface module. Through the RS422 serial communication protocol, this realizes the data interaction with the end effector controller;
(3) AD interface module. This realizes eight channels of force/torque, five channels of temperature and three channels of analog reference voltage information collection from the elastomer. The original force/torque signal is amplified by the triple-instrument amplifier circuit, and the filtered signal input goes to the analog-to-digital conversion module to achieve analog-to-digital conversion;
(4) DA interface module. This realizes the compensation voltage and feeds it back to the output terminal of the electric bridge to compensate the zero drift of the F/T sensor.

## 4. Space Environment Adaptability Design of F/T Sensor

### 4.1. Mechanical Vibration Design

**Figure 14:**
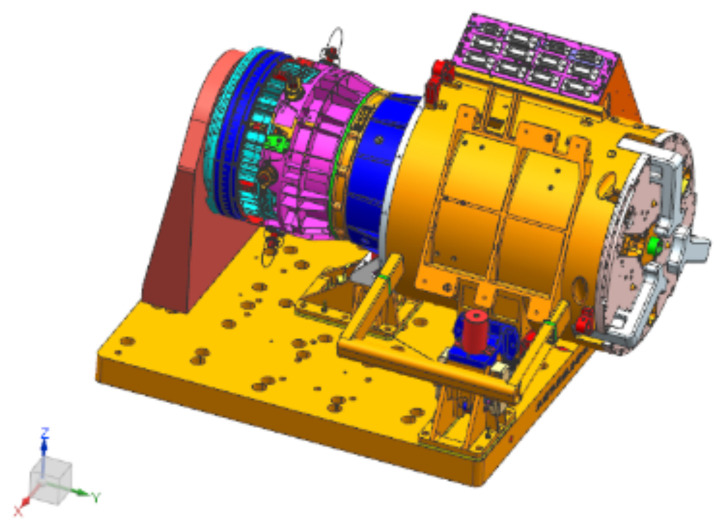
The 3D model and coordinate system definition.

As part of the EMM, the F/T sensor needs to carry a rocket into space with the EM. Therefore, the F/T sensor will be subject to mechanical vibration as it enters space. Therefore, mechanical vibration design must be considered. Figure 14 shows the three-dimensional model and coordinate system definition of the EE alone in the vibration test.

The mechanical analysis model construction was founded on the following principles: (1) in the key force transmission path, the mesh is dense; (2) the non-critical parts are simplified based on the principle of equivalent energy and stiffness.

**Figure 15:**
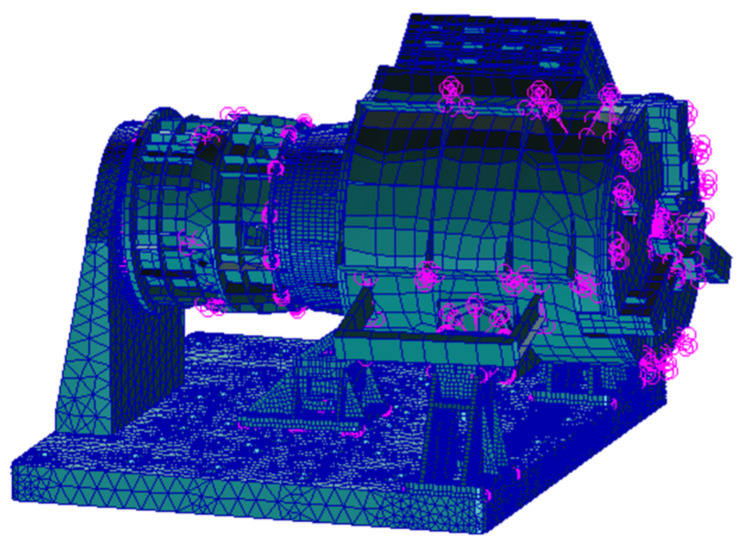
The finite element model of the EE and F/T sensor.

According to the above principles, shell elements and solid elements are used as structural elements when modeling. The finite element model and restraint positions of the EE are shown in Figure 15.

### 4.2. Thermal Control Design

According to the orbital characteristics of the CSS, the working mode of the EMM, the position of the heat dissipation surface, the performance of the thermal control coating and the temperature requirements of each component, a combination of passive thermal control and active thermal control can use to achieve thermal protection of the F/T sensor.

#### 4.2.1. Passive Thermal Control

The outer surface of the F/T sensor shell is covered by 20-unit multi-layer insulation components and the mask uses an external flame-retardant cloth. The housing of the F/T sensor and the bottom flange of the EE are heat conductors.

#### 4.2.2. Active Thermal Control

The F/T sensor shell and the EE are equipped with a heating zone. The main loop of the heating circuit is controlled by a temperature relay; the backup loop is controlled by temperature relay and a thermistor sensor. All thermistor sensors are arranged on the EE. The layout of the heating element of the F/T sensor is shown in Figure 2.

### 4.3. Anti-Radiation Design

The F/T sensor mainly realizes the acquisition of force/torque information and temperature information, which integrates semiconductor electronic components, digital logic circuits, etc.; therefore, radiation protection design is required.

#### 4.3.1. Protection Design of Total Ionization Dose Effect

The F/T sensor has one data-acquisition circuit board with a thickness of 2.0 mm, inside the sensor, protected by a thick shell, and the environment is relatively good. The thickness of the F/T sensor housing is 4 mm titanium alloy. From the above radiation shielding analysis, the internal radiation dose D_0_ of the electronic product under the shielding can be determined. According to the total dose resistance data (D_1_) of the components and materials in the sensor, and the internal dose value (D_0_) of the electronic product, the satisfaction of the total dose effect requirement is obtained. The criteria are as follows: (1) if D_1_/D_0_ ≥ RDM, it satisfied the requirement of the total anti-ionization dose; (2) if D_1_/D_0_ < RDM, the total anti-ionization dose requirement is not satisfied, and the protection design must be applied.

#### 4.3.2. Single-Event Effect Protection

(1) Single-event upset (SEU) protection
  The main measures are as follows:
    1. Select FPGA with FLASH structure;
    2. Optimize circuit structure.
(2) Single-event latch effects (SEL) protection
  1. A resistance-capacitance network is designed at the power input of the circuit board, a series of current-limiting resistors, and a decoupling tantalum capacitor are used to prevent the power supply from triggering lock due to overvoltage.
  2. In terms of component selection, anti-locking chips, such as CPU, RAM, PROM, etc., are preferred for important and key components.
  3. We designed a power supply circuit with the function of preventing SEL to realize the protection of the chip.
  4. Using multi-layer printed boards, the power supply and ground each occupy one layer, which improves the power supply loop.
  5. We placed a filter capacitor near the power terminal of the CMOS device. The external signal input terminal does not connect to the CMOS device directly. The external signal CMOS output adopts diode isolation. The input floating pins of all CMOS devices are grounded or connected to the power supply.

## 5. Manufacture of F/T Sensor

**Figure 16:**
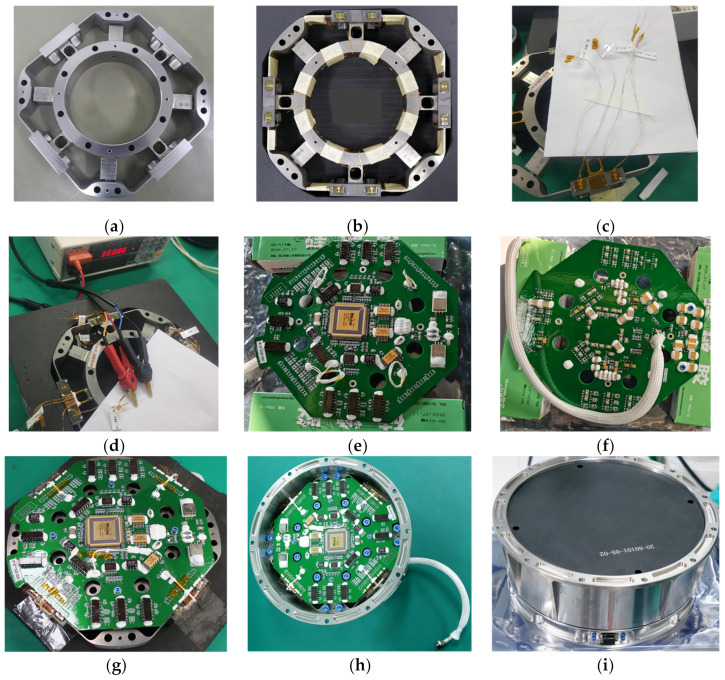
The main manufacturing process of the F/T sensor (**a**–**i**).

The manufacturing process of the F/T sensor affects the performance of the sensor directly. It includes the following steps: (1) processing of elastomer, (2) strain gauge paste and wire bonding, (3) strain gauge numbers and Wheatstone bridges building, (4) strain gauge resistance measurement, (5) Wheatstone bridges trimming, (6) electrical system processing, (7) debugging and preventions, (8) electrical systems and elastomers strain gauge electromechanical integration, (9) debugging and (10) sensor assembly. The main manufacturing process of the F/T sensor is shown in Figure 16.

## 6. Calibration of F/T Sensor

The calibration of the F/T sensor has a significant effect on the measurement accuracy. It is key to carry out the calibration experiment and calculate the calibration matrix, which reflects the characteristics of the F/T sensor. Suppose the F/T sensor is a linear system, then the output voltage of each Wheatstone bridge and the corresponding force/torque applied to the origin of the sensor coordinate system has a linear relationship, and the expression is as follows:

***U*** = *A**F*** + ***B*** (12)

In Equation (12), ***U***∈*R_n × 1_* is the Wheatstone bridge output vector of the F/T sensor; *A*∈*R_n × m_* is the F/T sensor calibration matrix; ***F***∈*R_m × 1_* is the generalized force vector; ***B***∈*R_n × 1_* is the intercept of the sensor; *m* is the dimensional of measuring the generalized force and *n* is the number of Wheatstone bridges.

### 6.1. Calibration F/T Sensor by Experiment

**Figure 17:**
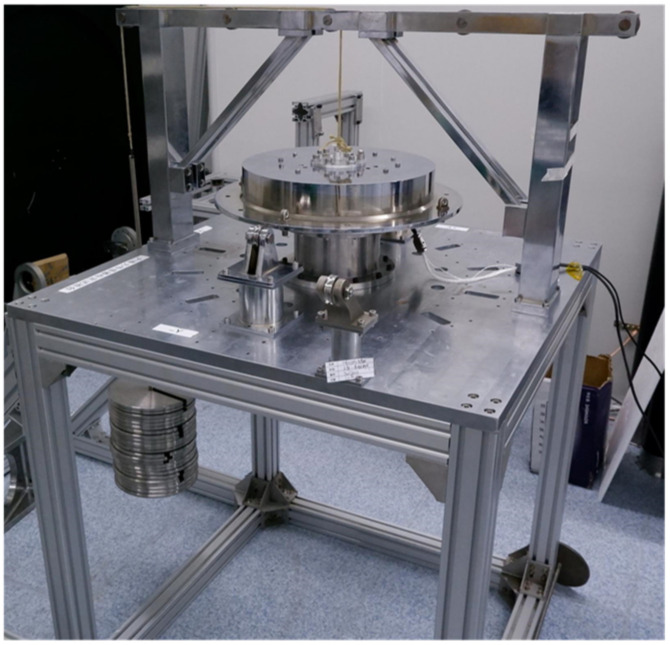
Calibration testbed.

The sensor is calibrated by a pulley weight system, which is shown in Figure 17.

**Figure 18:**
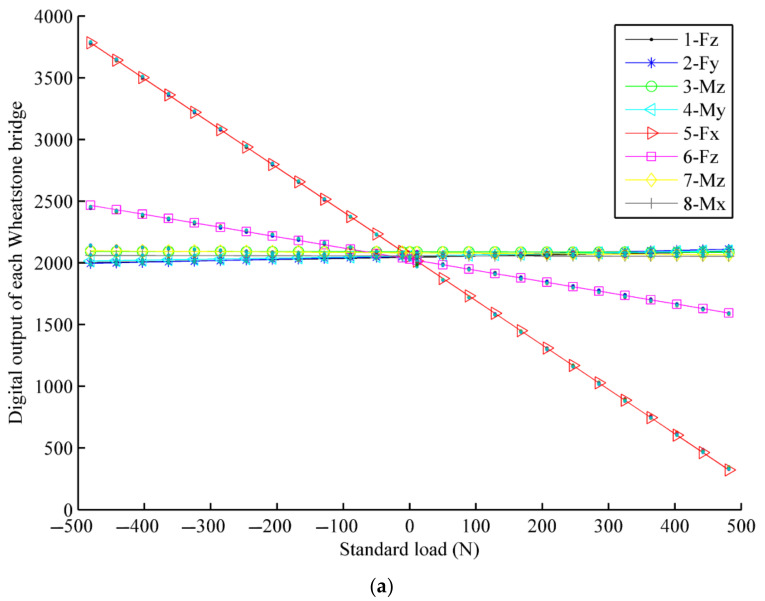

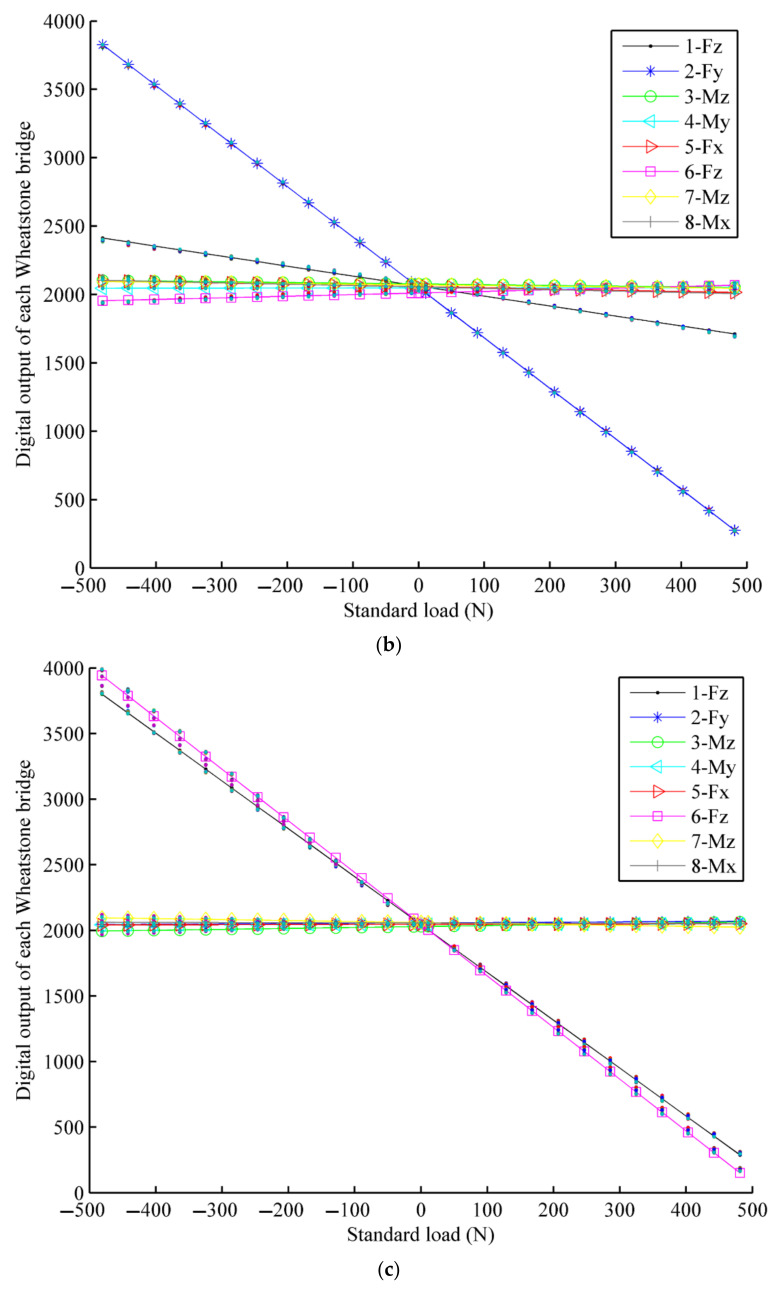

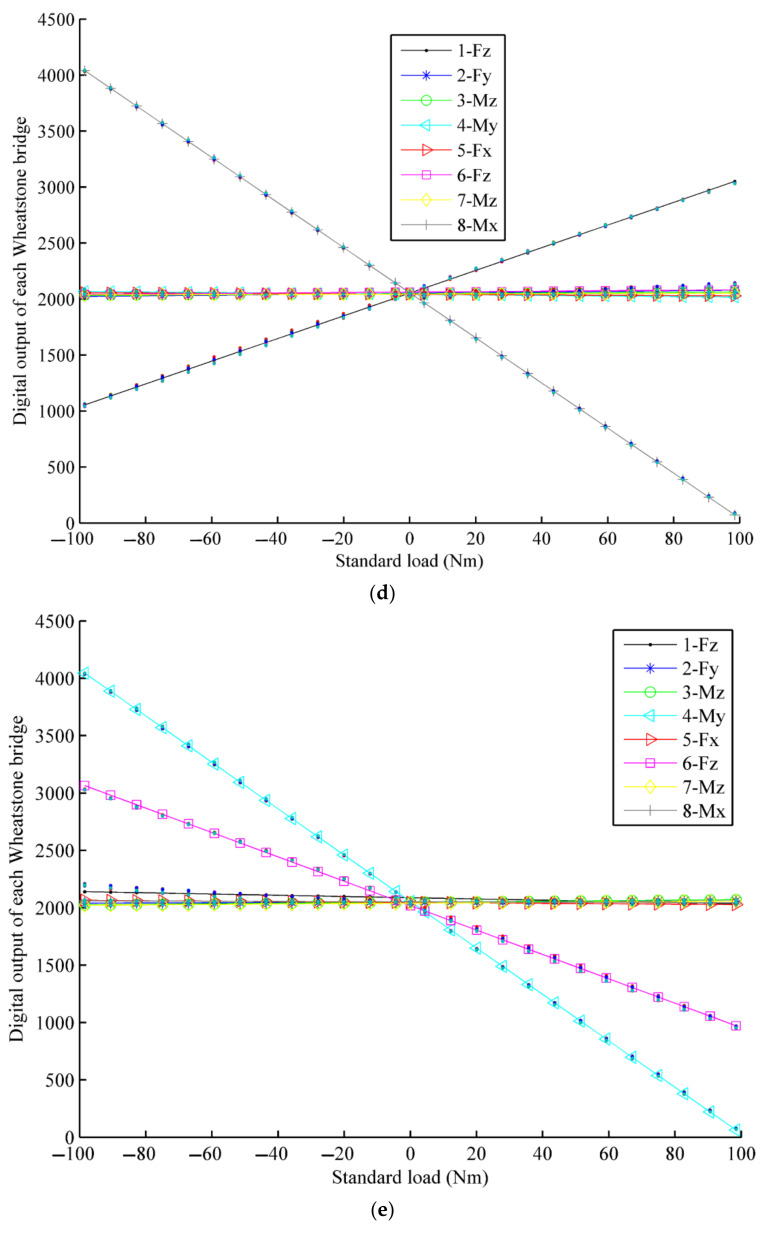

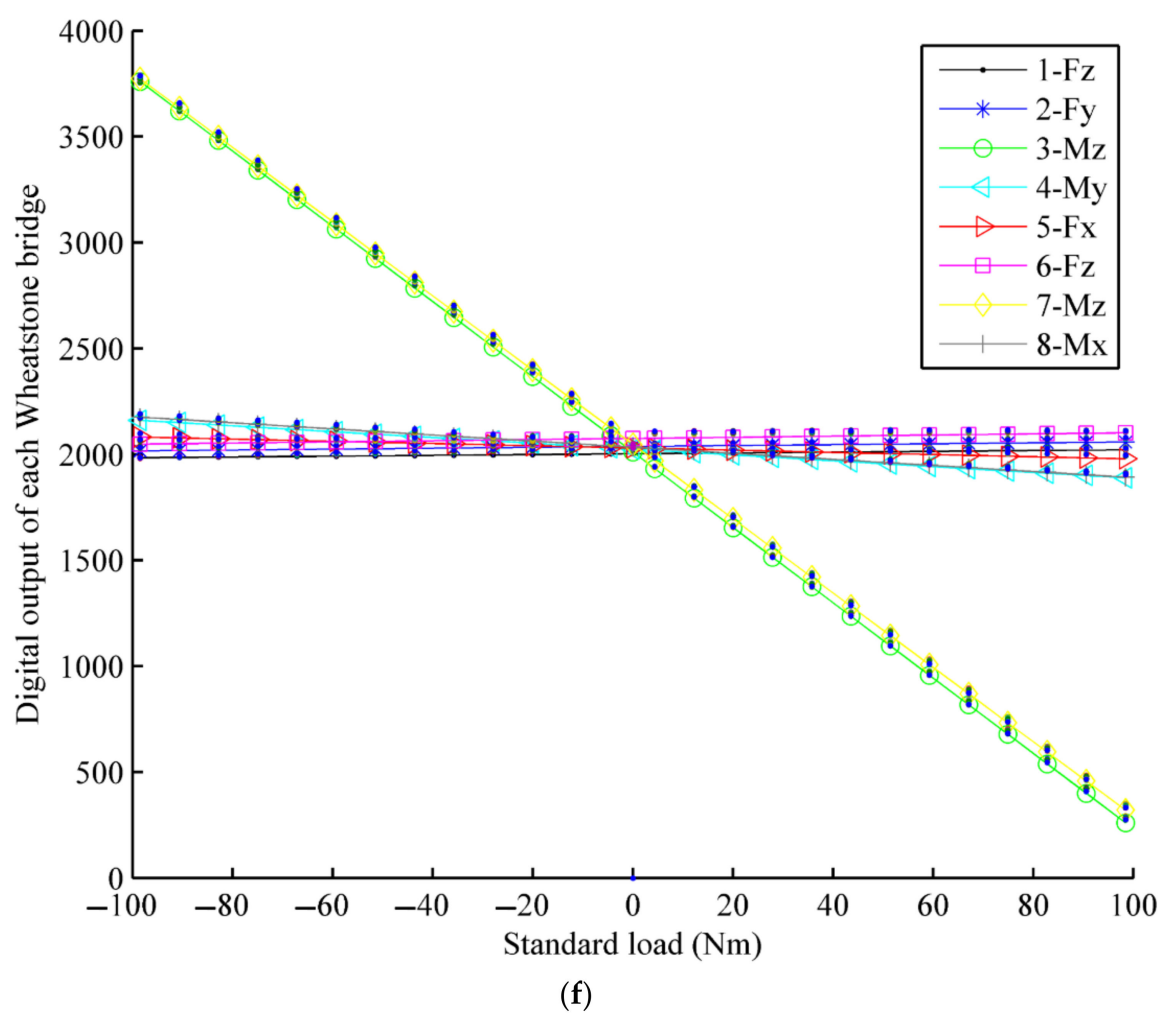
The calibration of F/T sensor. (**a**) The calibration of force *Fx*; (**b**) The calibration of force *Fy*; (**c**) The calibration of force *Fz*; (**d**) The calibration of *Mx*; (**e**) The calibration of *My*; (**f**) The calibration of *Mz.*

The calibration experiment results by least squares method are shown in Figure 18.

By processing the experimental data of the F/T sensor, the performance of the F/T sensor developed can be seen, as shown in Table 1. The following conclusions can be obtained from Table 1: the linearity of the sensor is less than 3%, the maximum linearity is 2.60% in the *Fz*, and the minimum linearity is 0.56% in the *Fy*; the maximum hysteresis characteristic of the sensor is 1.4% of the *Fz*, and the minimum hysteresis characteristic of the sensor is 0.51% of the *My*; the stability of the sensor is good, and the maximum stability error is 0.39% of the *Fz*; the maximum repeatability error is 1.72% in the *My*, the minimum repeatability error is 0.49% of the Fx; the accuracy of the sensor is high, the maximum accuracy is 1.18% in the *Fz*, and the minimum accuracy is 0.49 % in the *Mx.*

It can be seen from Table 1 that the sensor has good linearity, hysteresis, stability, repeatability and the accuracy. In addition, the *Fz* direction has the worst linearity, hysteresis and accuracy.

The sensor coupling errors obtained are shown in Table 2. It can be seen from Table 2 that the maximum coupling error in the *Fx* is −1.81%; the maximum coupling error in the *Fy* is −1.15%; the maximum coupling error in the *Fz* is 0.54%; the maximum coupling error in the *Mx* is −0.78%; the maximum coupling error in the *My* is 0.59% and the maximum coupling error in the *Mz* is 1.24%.

### 6.2. Error Analysis of Strain Simulation and Experimental Test

According to the F/T sensor experimental test, the test measurement strain can be obtained; then, the error between the simulation and the measurement will be calculated. The strain simulation results are shown in Figure 8, Figure 9, Figure 10 and Figure 11. Strain measurement and error are shown in Table 3. It can see from Table 3 that the absolute errors between the measurement strains and the simulation strains are less than 10%, indicating that the simulation results are accurate and credible.

## 7. Sensor Space Environmental Adaptability Test

### 7.1. Mechanical Vibration Test

#### 7.1.1. Measuring Acceleration Sensor Layout

**Figure 19:**
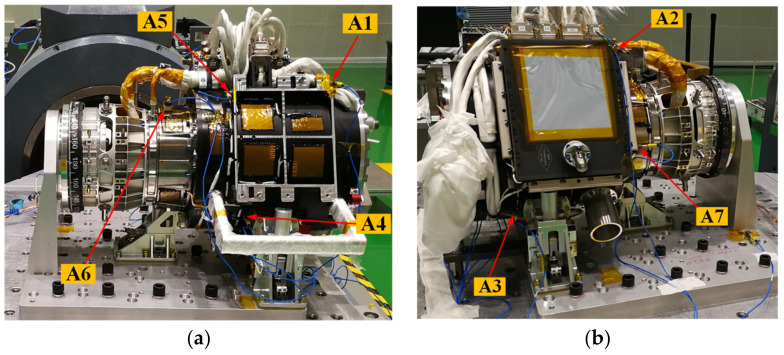
Measuring acceleration sensor position. (**a**) Position of measuring acceleration sensor A1, A4, A5, A6; (**b**) Position of measuring acceleration sensor A2, A3, A7.

The EE and F/T sensor are installed with seven acceleration points in the vibration test, which are all three-axis acceleration sensors, and the direction is consistent with the global coordinate system. The specific locations of the measuring points are shown in Figure 19.

#### 7.1.2. Sinusoidal Vibration Test Conditions

The sinusoidal vibration conditions are shown in Table 4.

#### 7.1.3. Random Vibration Test Conditions

The conditions of random vibration are shown in Table 5.

#### 7.1.4. Vibration Test Results

The sinusoidal vibration test data are shown in Table 6. It can see that the maximum response of sinusoidal vibration acceleration is 13.00 g, magnified 3.33-times, which occurred at measuring point A2 in the Y direction.

The random vibration test data are shown in Table 7. It can be seen that the maximum response of random vibration is 20.05 grms, and the magnification is 3.08-times, which occurs at the measuring point A1 in the X direction.

### 7.2. Thermal Vacuum Environment Test

The thermal vacuum test is a must-do test for aerospace products. It is used to test the working performance of the product in a vacuum environment and under high and low temperature changes.

#### 7.2.1. Thermal Vacuum Test Conditions

The thermal vacuum test conditions are as follows:

(a) Environmental pressure: no more than 1.3 × 10^−3^ Pa;
(b) Test temperature: The temperature range is −35 °C~+55 °C. The temperature of the heat sink under low-temperature conditions is lower than −70 °C, and the temperature of the heat sink under high-temperature conditions is higher than +20 °C;
(c) Average temperature change rate: not less than 1 °C/min; temperature change rate should be at least >0.5 °C/min (according to equipment capability);
(d) Number of cycles: 3.5 cycles for main part and 2 cycles for backup;
(e) Reference point test temperature tolerance: low temperature is 0 °C~−4 °C and high temperature is +4 °C~0 °C.

##### 7.2.2. Load Simulation Device of Thermal Vacuum Test

**Figure 20:**
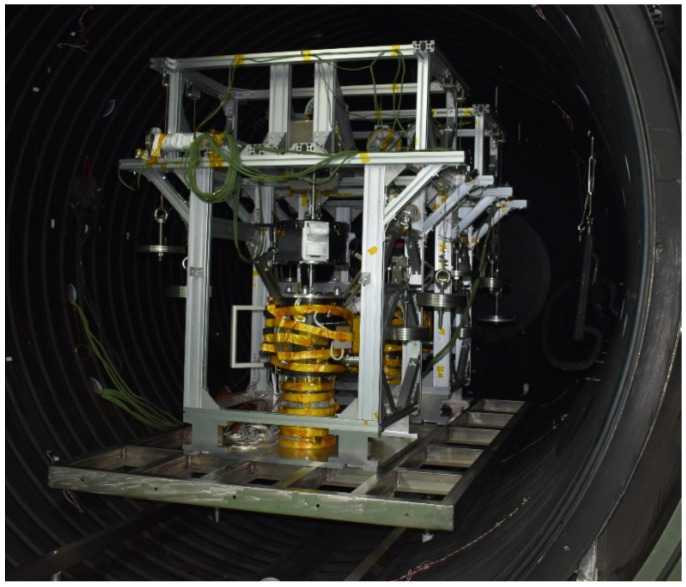
Load simulation device of thermal vacuum test of EE and F/T sensor.

The load simulation device for the thermal vacuum test is shown in Figure 20. The thermal vacuum test of the EE and F/T sensor needs to test the two products, in terms of the axial force and the lateral force of the EE. The axial force and lateral force of the device can be loaded with a load of 100 N, applied by weights and fixed pulleys, independently. Under the action of the F/T sensor, the lateral force and axial force of the end effector can be tested during the whole movement. The device can realize the thermal vacuum test of the EE and the F/T sensor.

##### 7.2.3. Thermal Vacuum Test Results

**Figure 21:**
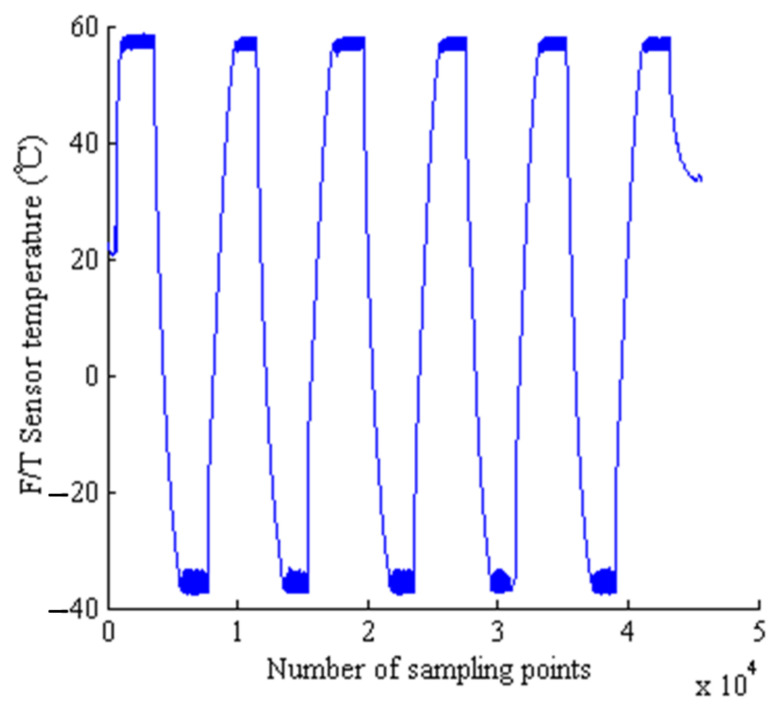
Temperature curve of sensor body.

The acceptance-level thermal vacuum test of the F/T sensor and EE has 5.5 cycles. The Thermal vacuum test results of the F/T sensor are shown in Figure 21. Across all 5.5 cycles, the minimum temperature of the sensor is −37.37 °C, and the maximum temperature is 57.1 °C.

##### 7.2.4. Force/Torque Information during Grasping and Releasing Progress of EE

**Figure 22:**
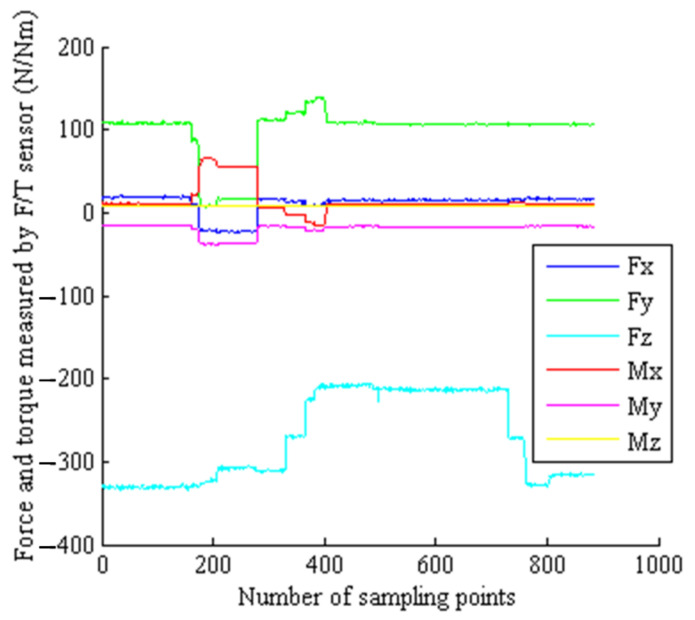
Six-dimensional force/torque information during the operation of the end effector grasping and releasing the target adapter.

Force/torque information during grasping and releasing progresses the target adapter of EE, measured by the F/T sensor, as shown in Figure 22. The test results show that the thermal vacuum test device can effectively complete the thermal vacuum test of the F/T sensor and the EE. The F/T sensor meets the thermal vacuum environment requirements of EMM on-orbit application.

## 8. Conclusions

This paper focused on the development of the F/T sensor for the EMM of the CSS. The F/T sensor is made of a titanium alloy material, and the strain analysis is based on the analytical method and finite element method. Furthermore, the electrical system mainly accomplished the information acquisition of force/torque, AD, temperature, analog voltage, DA setting and RS422 serial communication. In view of the adaptability of the space environment, the F/T sensor was designed with mechanical vibration, thermal control and anti-radiation properties. Subsequently, the manufacturing process of the sensor is explained in detail. Then, a calibration test is carried out, and the test results showed that the developed F/T sensor has good linearity, hysteresis, stability, repeatability, accuracy and coupling error. Finally, a mechanical vibration test and a thermal vacuum test were carried out. The test results showed that the developed sensor meets the requirements and has the ability for on-orbit application.

## Figures and Tables

**Table 1:** The static performance of F/T sensor.

Index	*Fx*	*Fy*	*Fz*	*Mx*	*My*	*Mz*
Range(N/Nm)	±500	±500	±500	±100	±100	±100
Linearity	1.25%	0.56%	2.60%	0.92%	0.91%	1.63%
Hysteresis	0.93%	0.73%	1.42%	0.63%	0.59%	1.07%
Stability	0.10%	0.10%	0.39%	0.10%	0.10%	0.10%
Repeatability	0.49%	1.07%	0.82%	1.41%	1.72%	1.17%
Accuracy	0.75%	0.53%	1.18%	0.49%	0.53%	0.84%

**Table 2:** The coupling error of F/T sensor.

	Coupling Error (%)					
	*Fx*	*Fy*	*Fz*	*Mx*	*My*	*Mz*
*Fx*	-	−0.33	−0.06	0.07	0.35	0.23
*Fy*	−0.27	-	−0.32	−0.02	−0.33	1.24
*Fz*	0.50	0.54	-	−0.78	−0.26	0.05
*Mx*	0.02	0.36	−0.36	-	0.59	−0.20
*My*	−0.32	0.34	−0.14	−0.08	-	0.06
*Mz*	−1.81	−1.15	0.54	0.43	0.48	-

**Table 3:** Error between strain simulation and experimental test.

	Bridge 1-*Fz*	Bridge 2-*Fy*	Bridge 3-*Mz*	Bridge 4-*My*	Bridge 5-*Fx*	Bridge 6-*Fz*	Bridge 7-*Mz*	Bridge 8-*Mx*
Simulation	384.8 μ	1014.2 μ	1376.5 μ	1690.3 μ	1014.2 μ	384.8 μ	1376.5 μ	1690.3 μ
Test	365.4 μ	948.0 μ	1269.9 μ	1743.8 μ	924.0 μ	394.4 μ	1252.8 μ	1736.7 μ
Error	−5.04%	−6.53%	−7.74%	3.17%	−8.89%	2.49%	−8.99%	2.75%

**Table 4:** Sinusoidal vibration test conditions.

Parameter Name			Reference					
			Frequency Range (Hz)					
			5~10	10~17	17~25	25~45	45~75	75~100
Amplitude 0-p	X, Y, Z direction	Acceptance level	8.5 mm	3.4 g	4.0 g	9.5 g	3.0 g	3.9 g
Load scan rate		Acceptance level	4 oct/min					
Loading direction			Three axis					

**Table 5:** Random vibration test conditions.

Parameter Name		Reference			
		Frequency Range (Hz)			Total Root Mean Square(grms)
		10–250 Hz	250–600 Hz	600–2000 Hz	
Power Spectral Density	Acceptance level (X,Y,Z)	6 dB/Oct	0.06 g^2^/Hz	−9 dB/Oct	6.5 grms
Duration of each direction	Acceptance level	60 s			
Loading direction		Three axis			

**Table 6:** Sinusoidal vibration response data.

Direction	Measuring Point	Acceptance Grade Sine		Direction	Measuring Point	Acceptance Grade Sine		Direction	Measuring Point	Acceptance Grade Sine	
		Frequency (Hz)	Acceleration (g)			Frequency (Hz)	Acceleration (g)			Frequency (Hz)	Acceleration (g)
X	A1	44.98	10.39	Y	A1	99.72	12.34	Z	A1	34.51	10.72
	A2	44.98	10.25		A2	99.72	13.00		A2	34.66	10.31
	A3	44.98	9.76		A3	43.75	9.76		A3	34.71	11.19
	A4	44.78	9.74		A4	44.98	9.80		A4	34.46	10.20
	A5	45.11	10.26		A5	99.72	11.80		A5	34.31	10.06
	A6	44.98	10.15		A6	99.72	10.93		A6	34.61	9.94
	A7	44.98	9.91		A7	44.72	10.27		A7	34.71	10.15

**Table 7:** Random vibration response data.

Direction	Measuring Point	Direction	Acceptance Level Random	Direction	Measuring Point	Direction	Acceptance Level Random	Direction	Measuring Point	Direction	Acceptance Level Random
			Root Mean Square Acceleration (grms)				Root Mean Square Acceleration (grms)				Root Mean Square Acceleration (grms)
X	A1	X	20.05	Y	A1	X	14.63	Z	A1	X	16.71
		Y	6.49			Y	8.12			Y	9.04
		Z	9.69			Z	12.10			Z	11.80
	A2	X	11.15		A2	X	5.42		A2	X	8.49
		Y	6.39			Y	7.30			Y	7.85
		Z	4.92			Z	5.31			Z	11.04
	A3	X	8.81		A3	X	7.48		A3	X	9.13
		Y	3.81			Y	8.24			Y	6.41
		Z	7.20			Z	7.12			Z	8.40
	A4	X	9.40		A4	X	7.31		A4	X	8.07
		Y	4.34			Y	7.93			Y	5.53
		Z	6.58			Z	5.53			Z	7.86
	A5	X	9.37		A5	X	4.79		A5	X	7.31
		Y	4.29			Y	5.25			Y	5.05
		Z	6.22			Z	5.51			Z	10.05
	A6	X	8.52		A6	X	4.32		A6	X	7.22
		Y	6.38			Y	8.87			Y	9.11
		Z	3.85			Z	5.74			Z	11.98
	A7	X	7.31		A7	X	3.95		A7	X	5.57
		Y	4.97			Y	8.24			Y	7.34
		Z	5.25			Z	5.76			Z	12.60

## Data Availability

Not applicable.
